# Metastatic colorectal carcinoma to the right atrium: a case report and review of the literature

**DOI:** 10.1186/s40959-021-00108-9

**Published:** 2021-05-31

**Authors:** Humaira Sarfraz, Abeer Arain, Mukul K. Divatia, Mary R. Schwartz, Kirk E. Heyne

**Affiliations:** 1 Houston Methodist Hospital, 6565 Fannin St, Smith Suite 1001, Houston, TX 77030 USA; 2 Houston Methodist Cancer Center, Houston, TX 77030 USA; 3 Houston Methodist Hospital: Pathology and Genomic Medicine, Houston, TX 77030 USA

**Keywords:** Cardiac metastasis, Colon cancer, Next generation sequencing

## Abstract

**Background:**

Cardiac metastasis due to colon cancer is extraordinarily uncommon. Given the rarity of diagnosis, there is paucity of evidence and hence, no established guidelines for evaluation or clinical management strategy.

**Clinical presentation:**

We present the case of a 59 year old male with a previously treated colonic carcinoma who presented with new onset exertional dyspnea. He was noted to be having a right atrial mass on an echocardiogram performed at his cardiologist’s office. Further workup with CT angiogram of the chest confirmed a right atrial mass measuring 4.0 cm. Serum CEA was normal. Biopsies of the right atrial mass demonstrated metastatic moderately differentiated colonic adenocarcinoma. Mismatch repair protein expression analysis by immunohistochemistry showed no loss of MLH1, MSH2, MSH6 or PMS2 expression. Next generation sequencing for RAS and BRAF mutations was negative. Patient received treatment with FOLFIRINOX/ bevacizumab with noted reduction in size of mass.

**Conclusion:**

To the best of our knowledge, this is the first report of next generation sequencing results available on a biopsy of metastatic colorectal cancer to the heart with the largest literature review of 31 reported cases of metastatic colorectal cancer to the heart. It will help direct clinical management and also adds evidence to the potential efficacy of treatment of this rare aggressive disease with chemotherapy in combination with VEGF inhibitors.

## Introduction

Colorectal carcinoma is the third most common malignancy and the third most common cause of cancer deaths in both men and women in the United States [1]. The most common sites of metastasis of colorectal cancer are lymph nodes, liver and lungs, via lymphatic or hematogenous spread. Cardiac metastasis with colorectal cancer is rare, with very few cases reported in literature. Hence there is paucity of data regarding the best imaging modalities and treatment options for this condition.

Here we report a rare case of colorectal cancer metastatic to the heart, discovered 5 years after diagnosis and treatment of a primary colon cancer that responded remarkably to chemotherapy. We also provide the largest review of literature of cardiac metastases from colorectal carcinoma.

## Case

A 59 year old male presented with a 2 week history of progressively worsening exertional dyspnea (NYHA class II), bilateral lower extremity edema and abdominal distension. He denied any fever, chills, chest pain, cough, orthopnea, paroxysmal nocturnal dyspnea, palpitations, nausea, vomiting, abdominal pain, syncope or weight loss. He presented to the Emergency Room after his cardiologist found a right atrial mass on echocardiogram during an office visit. His past medical history was significant for treated colorectal cancer, hyperlipidemia and gout.

The patient was diagnosed with colonic cancer 5.5 years earlier for which he underwent surgical resection with right hemicolectomy. The patient did not receive any adjuvant chemotherapy or radiation. He continued to follow up with his primary oncologist with surveillance CT scans and had completed his 5 year surveillance.

Family history was significant for colon cancer in his uncle and breast cancer in two of his sisters. He denied any tobacco use or alcohol intake.

The physical examination was remarkable for decreased lung sounds at the lung bases, moderate ascites and bilateral 1+ pitting edema up to the knees. EKG and cardiac enzymes were unremarkable. The patient underwent a CT angiogram of the chest which revealed a 4.0 cm low attenuating right atrial mass. The lung parenchyma had some ground-glass opacities bilaterally which were thought to be related to motion artefact or less likely suggestive of pulmonary edema. Deep venous thrombosis was ruled out with a negative lower extremity Doppler. The following day, he underwent paracentesis which showed a Serum Ascitis Albumin Gap of 1.4 and 726 nucleated cells (41% lymphocytes, 54% macrophages and 5% neutrophils). Cytologic examination was negative for malignancy, with findings notable for mesothelial cells and macrophages. Cardiac MRI showed a 8.3 cm × 6.5 cm right atrial mass extending from the suprarenal IVC into the hepatic veins and occupying the majority of the right atrium. The mass was noted to be isointense to myocardium on T1 weighted imaging, hyperintense to myocardium on T2 weighted imaging; with positive uptake of Gadolinium contrast in the peripheral segments of the tumor on first pass perfusion imaging, and had heterogeneous uptake on Late Gadolinium Enhancement imaging. A small circumferential pericardial effusion was also seen. Other laboratory studies were majorly unremarkable; with a normal CBC, CMP, PSA and serum CEA levels. The major differentials at this point were atrial myxoma, sarcoma or metastatic disease. It was believed that the shortness of breath was liked related to the increased right atrial pressures.

**Fig. 1:**
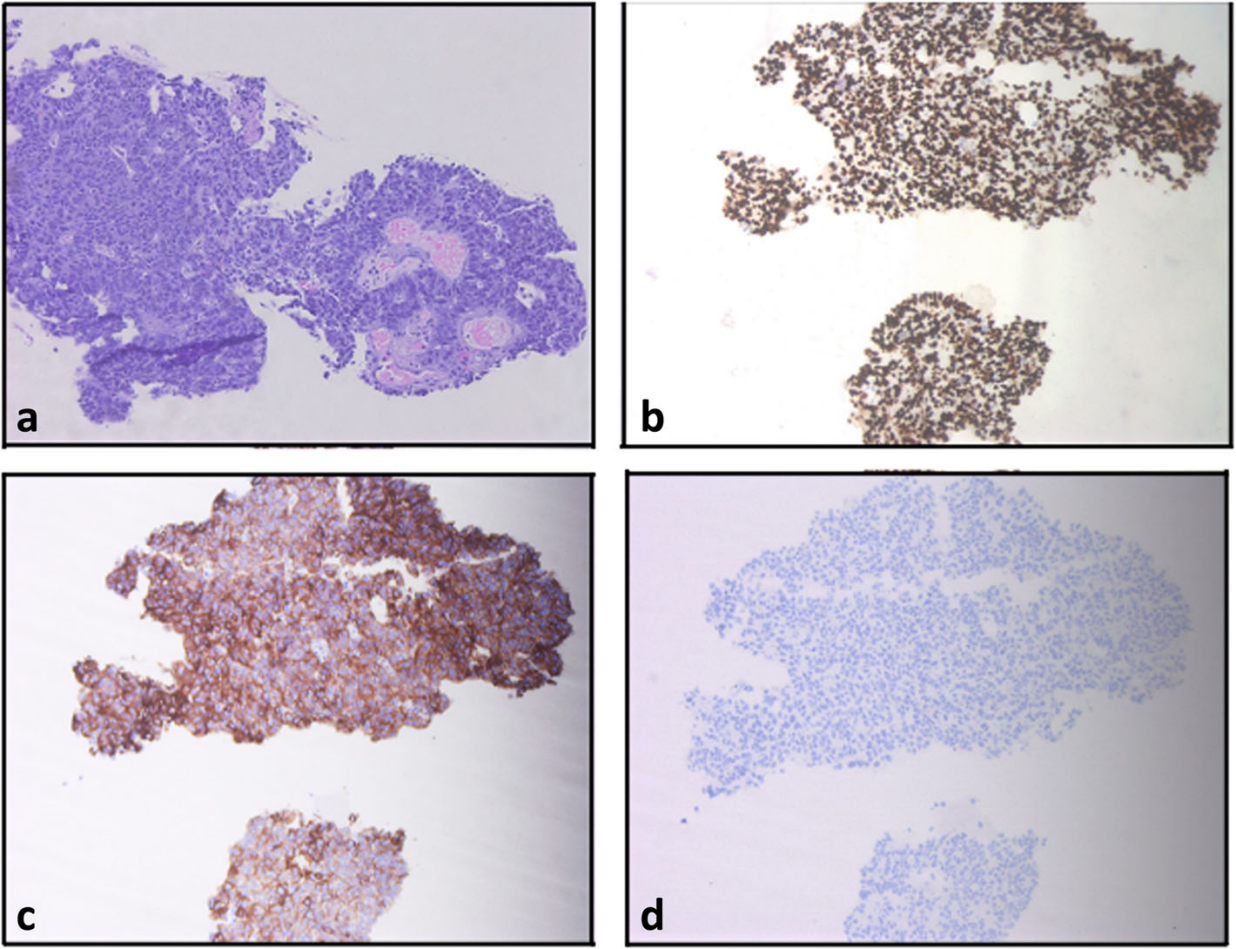
**A**: Metastatic moderately differentiated colonic adenocarcinoma with cribriform glandular growth pattern (H &E, ×100). **B**: Enteric differentiation demonstrated by strong immunohistochemical expression of CDX2. **C**: enteric differentiation demonstrated by strong immunohistochemical expression of cytokeratin 20(immunoperoxidase, × 100). **D**: negative cytokeratin 7 immunostaining (immunoperoxidase, × 100). Endomyocardium was not included in this spe cimen

Cardiothoracic surgery performed an echocardiographic and fluoroscopic guided biopsy of the right atrial mass. The biopsy showed metastatic moderately differentiated colonic adenocarcinoma (Fig. 1). Mismatch repair protein expression analysis by immunohistochemistry showed no loss of MLH1, MSH2, MSH6 or PMS2 expression. No RAS, HRAS, NRAS or BRAF mutation was detected on next generation sequencing. MRI of the brain was negative for metastasis. However, MRI bone scan showed that the patient had a low T1 signal intensity in the left distal clavicle that was suspicious for metastatic disease. PET/ CT, was performed to evaluate if the disease was oligometastatic, confirmed metastatic disease in the left distal clavicle with a pathological fracture. The right atrial mass was also noted on PET/CT with abnormal uptake (SUV of 11). Treatment was initiated with plans for 4 cycles of FOLFIRINOX/ bevacizumab. Radiation therapy to the distal clavicle was deferred at this point pending response to chemotherapy.

Colonoscopy was performed during the hospitalization to rule out a second primary colorectal carcinoma. It showed a patent end to side ileo-colonic anastomosis with no endoscopic evidence of colon cancer was noted in the entire colon.

On his clinic visit to receive his second cycle of chemotherapy, he was noted to be doing much better with his symptoms of dyspnea and lower extremity edema being significantly better. However, he noted that the left clavicle was a little more painful, so oral opioids were added. At this point, radiation to the clavicle was planned. Repeat echocardiogram performed a month later showed a decrease in the size of the right atrial mass from 4 cm to 3 cm. On his most recent clinic visit, he continues to be asymptomatic and tolerated cycle 7 of chemotherapy with bevacizumab. A cardiac MRI is pending for him with plans to attempt surgical removal by the cardiothoracic surgery team if the tumor has regressed to the supra-diaphragmatic area.

## Discussion

Cardiac metastasis are uncommon. Literature reviews for cardiac metastasis dating from 1948 to 2007 show that the incidence of cardiac metastasis ranges between 2.3–18.3% [2]. The highest incidence was noted by Hanfling in 1960 who reported an autopsy series comprising of 127 cases of cardiac metastasis in cancer patients with an incidence of 4.8% of all autopsies and 18.3% of all cancer related deaths. More recent studies from 2005 to 2007 show an incidence of 2.3–9.1%. An important consideration is that cardiac metastasis may be underestimated due to being clinically silent [2, 3]. The postulated mechanisms of cardiac metastasis include direct extension, hematogenous dissemination, lymphatic spread or by intra-cavitatary spread via the superior/ inferior vena cava. The highest rates of cardiac metastasis have been reported with pleural mesothelioma, melanoma and lung [2].

On our literature review, we found a total of 31 cases of metastatic cardiac involvement secondary to colorectal cancer. The patients ranged from 41 to 81 years with a median age of 70 years at diagnosis of metastasis. There is an overall male preponderance with 21 males vs 10 females reported thus far. Interval between initial diagnosis of malignancy and discovery of cardiac metastasis appears to be very wide. Upon literature review, the interval appears to extend unto 17 years after initial diagnosis of colorectal malignancy. Expert consensus regarding duration of surveillance for colorectal cancer per ASCO: American Society of Clinical Oncology, CCO: Cancer Care Ontario, NCCN: National Comprehensive Cancer Network ranges between roughly 3–5 years post initial diagnosis and treatment.

When symptomatic, cardiac metastases usually present with clinical features of heart failure including dyspnea, palpitations, thromboembolism or tumor extension into the superior or inferior vena cava manifesting with edema or obstructive signs [4, 5].

The differential to be considered when evaluating cardiac masses includes malignancies, atrial myxomas, thrombi and vegetations. *Streptococcus gallolyticus* infective endocarditis has been associated with colorectal malignancies. Various imaging modalities including transthoracic/ transesophageal echocardiography, CT or MRI can be utilized to provide a comprehensive view of the lesion and identify potentially infiltrative cardiac tumors. Molecular profiling of the metastatic tumor may help tailor the therapy and should be considered as part of the workup. Although colonoscopy is part of the initial workup for primary colon cancer; it should also be considered in patients who present with metastatic colorectal cancer to evaluate for a second primary colorectal carcinoma, as done for our patient. Henceforth, it would be plausible to consider further evaluation for cardiac involvement in any patient with a history of malignancy presenting with cardiopulmonary symptoms.

Metastatic cardiac tumors are generally associated with an aggressive disease course and a generally poor outcome. Only 9 out of the 31 reported cases of metastatic colorectal carcinoma to the heart were alive at the time of the case being reported. However, given the latest developments in diagnostic and therapeutic modalities, increased awareness and higher accessibility to health care over the years; it has become easier to identify these rare metastases.

In the event that an isolated metastasis or oligometastatic disease is found; multiple treatment options including surgical resection and chemotherapy may be offered. However, the ultimate decision would be contingent upon disease volume, tumor burden, comorbidities, genetic profiling and individualizing risk versus benefits for the patient. Surgery may be considered in a case of solitary cardiac metastasis or as a debulking maneuver if the lesion causes hemodynamic instability to bridge to ultimate treatment with chemotherapy. Upfront chemotherapy may be the best treatment for other patients. There is no consensus on treatment of cardiac metastasis and further studies are required to outline optimal treatment strategies for these patients.

Amongst the various treatment modalities that were used in the various cases reported; 14 patients underwent surgery only, 3 underwent chemotherapy only, 4 had surgery followed by chemotherapy, 9 received no treatment, treatment was not available for 2 of the reported cases.

Treatment with chemotherapy only in these patients with metastatic colorectal cancer lead to variable response to treatment but the patients invariably had ultimate disease progression or recurrence. Choufani et al. reported a case of patient who was 16 months post completion of treated metastatic colorectal cancer to the liver. This patient presented with new onset abdominal distension and dyspnea on exertion and was noted to be having a right atrial mass along with progression of liver metastases, new findings of ascites and pleural effusions. He received four doses of Irinotecan monthly with complete resolution of right atrial mass and ascites. However, subsequent CT scans showed partial recurrence of the right atrial mass and rising CEA levels. Overall, the patient was doing well symptomatically 10 months after resumption of irinotecan [6]. Pontillo et al. reported a case of Right atrial mass noted incidentally as part of pre-operative cardiac evaluation. This was noted 7 years following the patient’s previous diagnosis and treatment for CRC. She was also noted to be having peritoneal carcinomatosis and was treated with standard medical therapy. No specific names or duration of treatment was mentioned [7]. Meanwhile the case reported by Tsuji involved an incidentally diagnosed RV tumor with colonoscopy confirming wild type KRAS on histopathological examination. The tumor was deemed inoperable by surgeons and the patient was found to be having metastatic lung involvement as well. The patient received 10 cycles of 5-fluorouracil, oxaliplatin plus panitumumab. Oxaliplatin was held thereafter due to neuropathy and was deemed to be in partial response after 12 courses (10 months after initial treatment). However, follow up CT after 15 cycles showed progressive disease in the heart and the patient eventually elected for palliative care but was alive 2 years after his diagnosis [8].

Amongst the four cases with surgery followed by chemotherapy, two patients received bevacizumab-based treatments. Bianchi et al. reported a case with hyper-metabolic focus noted on PET/ CT in the Right Atrium following mildly elevated CEA levels at 35 ng/ml on routine surveillance 2 years out of treatment completion for colorectal cancer. Patient underwent a minimally invasive thoracotomy with mass excision which revealed the metastasis. The patient received 1 cycle of leucovorin, fluorouracil and irinotecan (FOLFIRI) and bevacizumab but was unable to tolerate subsequent sessions. Patient died 3 months later due to massive pulmonary embolism [9]. However, since the patient received only one cycle, it is uncertain to assess whether the patient could have had potential response to treatment. The patient reported by Butler et al. was noted to be having a mass in right atrioventricular groove on a CT scan/ TTE following elevated CEA levels 17 years following her initial diagnosis and treatment. The patient underwent cardiac surgery and resection. Follow up PET/ CT in 6 months after surgery showed extension of neoplastic disease in her left and right atria. Gene markers reportedly showed sensitivity to chemotherapy; however specifics regarding which markers were checked is not mentioned. The patient was treated with FOLFOX only initially; then bevacizumab was added. And 2.5 years since diagnosis of cardiac metastasis, she was placed on single agent bevacizumab without disease progression [10]. This case was similar to ours with regards to choice of treatment agents and response noted. In another case report by Namireddy et al., the patient presented with shortness of breath and syncope; 1 year after treatment for T3N1 rectal adenocarcinoma and was found to be having right atrial mass and moderate pulmonary embolism. The patient underwent median sternotomy with wide excision of the right atrial wall and was started on chemotherapy but no comments were noted with regards to the choice of the regimen or patient’s response to treatment [11]. de la Fouchardière et al. reported a patient who was 3.5 years post diagnosis for rectal cancer, the patient’s CEA levels were found to be up trending which prompted a PET/ CT scan which showed a 6 cm × 3 cm mass in the RV. The patient underwent cardiac surgery with palliative resection and subsequent pathology report revealed an adenocarcinoma confirming rectal origin. A post-operative CT scan showed residual intracardiac mass with pericardial effusion. He received 6 cycles of FOLFIRINOX after which the heart mass was noted to be stable and the pericardial effusion decreased. The patient completed a total of 11 cycles of FOLFOX. Patient asymptomatic throughout [12] (Table 1).

**Table 1:** Cardiac metastases from colorectal cancer. Review of the literature

	Author	Age (years)/ Sex	Primary tumor site	Primary malignancy and stage	Time between initial diagnosis and discovery of cardiac metastasis (years)	Diagnostic modality of cardiac metastasis	Cardiac Site	Tumor size (cm)	Treatment	Outcome	Reference #
1	Henuzet (1982) [13]	60y/M	Rectum	NA	NA	TTE	RV	2	Resection	Dead	13
2	Nishada (1991) [14]	69y/M	Colon	Moderately differentiated adenocarcinoma; NA	0.66	TTE and MRI	RA	10 × 8 × 3	Resection	Died 2 wk. after surgery	14
3	Massachussetts General Hospital (1992) [15]	75y/M	Colon	NA	NA	Autopsy	RV	7.5 × 4.5 × 4	No	Dead	15
4	Parravicini (1993) [16]	47y/M	Rectum	NA	2	Surgery	RV	10 × 4 × 3.5	Resection	Dead	16
5	Testempassi (1994) [17]	71y/M	Colon	Stage III	NA	MRI	RV	NA	NA	NA	17
6	Zipoli (1994) [18]	41y/F	Colon	M adenocarcinoma; NA	NA	TTE	RA	4.1 × 3.7	Resection	Died 6mo after resection of tumors	18
7	Teixeira (1997) [5]	81y/M	Colon	Mucinous adenocarcinoma; T3N0M0 (Stage II)-Duke B2	5	TTE and CT	RA	NA	Supportive	Died shortly after diagnosis of RA tumor	5
8	Lord (1999) [19]	71y/M	Rectum	Dukes C	3	TTE	RV	NA	No	Dead	19
9	Choufani (2001) [6]	59y/M	Colon	Moderately differentiated adenocarcinoma; T3N1Mx (Stage III)	1.25	TTE	RA	5 × 3	Chemotherapy-Irinotecan	Alive at 10 mo from diagnosis of RA tumor	6
10	Koizumi (2003) [20]	65y/M	Colon	Well-differentiated adenocarcinoma; Stage III-Duke C	NA	TTE	RA	6 × 5	Resection	Lived for 11 mo after resection and without any chemotherapy	20
11	Oneglia (2005) [21]	70y/F	Colon	Duke C	NA	TTE, TEE	RV, Tricuspid valve	NA	Resection	Died a few hours after surgery	21
12	Lui (2004) [22]	71y/F	Rectum	Duke B	NA	TTE, CT scan, MRI	RV and RVOT	5 × 3.5	Resection	In hospital death	22
13	de la Fouchardière (2007) [12]	70y/F	Rectum	pT3N2	3.5	PET/ CT, TTE, CT scan	RV	6 × 3	Resection	Alive at 10 months from follow up on adjuvant chemotherapy	12
14	Moreno-Vega (2006) [23]	70y/F	Colon	pT3N2M1	NA	TTE, CT	RV	NA	Diagnostic pericardiocentesis	In hospital death	23
15	Choi (2009) [24]	70y/M	Colon	Moderately differentiated adenocarcinoma; T4N2M1 (Stage IV)	0	TTE	RA	5.5 × 5 × 3	Resection	Died 3 days postoperatively secondary to recurrent cardiac bleeding	24
16	Makhija (2009) [25]	70y/M	Rectum	Poorly differentiated mucinous adenocarcinoma; T3N2M1 (Stage IV)	0	TTE	RA	5.6 (greatest diameter)	Debulking due to invasion of tumor in RA wall	Alive 4.66 years from time of diagnosis for rectal cancer	25
17	Ngow (2012) [26]	59y/M	Colon	Dysplastic polyp in sigmoid colon with ascites/ multiple lung nodules/ ascites (no tissue confirmation of malignancy)	NA	TTE	RA	5 × 6	None	Died prior to therapy due to cardiac arrest	26
18	Patel (2012) [4]	72y/M	Colon	Moderately differentiated mucinous adenocarcinoma; T4bN2bM0 (Stage III)	3	TTE and TEE	RA	8.5 × 5 × 4	None	Sudden cardiac death secondary to pulmonary embolism related to malignancy	4
19	Butler (2012) [10]	77y/F	Rectosigmoid	Rectosigmoid well differentiated adenocarcinoma, T3N1 (Stage III) s/p surgical resection, adjuvant chemotherapy with 5-FU, levimasole and RT	17	CT	RA	NA	Surgery followed by chemotherapy	f/u PET/CT in 6 mo shows extension, treatment started with oxaliplatin with bevacizumab	10
20	Sudo (2013) [27]	70y/F	Colon cancer	NA (Article in Japanese)	NA	NA	RVOT	NA	Surgery	Living 2mo after	27
21	Mihali (2013) [28]	56y/M	Colon	Stage IV-metastatic to colon adenocarcinoma with adrenal, liver, bone, and mediastinal lymph nodes metastasis and paraneoplastic polymyositis	0	Autopsy	Diffuse micrometastases in myocardium	NA	NA	Death	28
22	Pontillo (2014) [7]	70y/M	Colorectal	Colorectal adenocarcinoma s/p L hemicolectomy and chemotherapy in remission	7	TTE and TEE	IVC into RA, also liver	NA	Standard medical therapy	Unknown	7
23	Reisenauer (2016) [29]	67y/M	Rectum	Stage IIIC adenocarcinoma	1	CT	Invasion into LA	7.6	Resection	Alive 1 mo post-discharge	29
24	Bianchi (2016) [9]	77y/F	Colon	Colonic adenocarcinoma-Dukes B cancer in the descending colon s/p surgery and 6mo chemotherapy	2	PET/ CT and TTE	Polylobular RA mass	4.1 × 3.5 × 3.5	Surgical excision followed by 1 cycle of FOLFIRI and bevacizumab	Death 3 months later due to pulmonary embolism	9
25	Kasama (2016) [30]	72y/M	Sigmoid colon	Stage IIIa later stage IV (metastatic to lungs)	NA	CT	RA	NA	Volume resection	Dead 3 months after surgery	30
26	Namireddy (2017) [11]	51y/M	Rectal	Rectal adenocarcinoma-T3N1aM0 s/p surgery and adjuvant chemotherapy	1	CT and TTE	RA	3.1 × 2.3	Surgery followed by systemic chemotherapy	NA	11
27	Ayyala (2017) [31]	69y/F	Rectal cancer	Rectal adenocracinoma s/p surgery and adjuvant chemotherapy	5	TTE	RA	4 × 3.5	None	NA	31
28	Tsujii (2017) [8]	76y/F	Colorectal	Well differentiated colorectal cancer (Stage IVb: cT2N0M1b)-metastatic to heart, lung-wild type KRAS	0	TTE, CT and MRI	RV	5.4 × 3.2 × 3.1	5-FU, oxaliplatin and panitumumab	Alive at 2 years after diagnosis	8
29	Elbatarny (2019) [32]	59y/M	Colon cancer	Colon cancer s/p surgery and chemotherapy	17	TTE and cardiac MRI	RV	Occupied 80% RV	Surgical debulking	NA	32
30	Graf (2019) [33]	70s y/M	Colon	Stage IV adenocarcinoma (metastatic to liver)	0	TTE and CT	RA/ RV/RVOT/Truncus pulmononalis microcavitations	microcavitations	Refused treatment	Death 4 mo after diagnosis	33
31	Current	59y/M	Colon	NA	5	TTE and cMRI	RA	8.3 × 6.5	Chemotherapy	Alive	

*Abbreviations*: *VEGF* Vascular Endothelial Growth factor, *TEE* transesophageal echocardiography, *TEE* transthoracic echocardiography, *IVC* Inferior Vena Cava, *MLH* MutL homolog, *MSH* MutS homolog, *NYHA* New York heart Association, *SAAG* Serum Ascitis Albumin Gradient, *CMP* Complete Metabolic Profile, *CBC* Complete Blood Count, *PSA* Prostrate Specific Antigen, *CEA* Carcinoembryonic Antigen, *FOLFIRINOX* leucovorin, fluorouracil, irinotecan, and oxaliplatin, *NA* Not Applicable

## Conclusion

Metastatic cardiac tumors have been linked with an aggressive disease course and poor outcomes in general. Given the recent developments in diagnostic and therapeutic modalities, integration of next generation sequencing; management options have evolved over the years for metastatic colorectal involvement of the heart. Hopefully, this literature review will help guide clinical management and also add to the evidence available. However, further studies are needed to formulate treatment strategies for this rare entity.

## Data Availability

Allowed.
